# Vascular measurements correlate with estrogen receptor status

**DOI:** 10.1186/1471-2407-14-279

**Published:** 2014-04-23

**Authors:** Mark C Lloyd, Khalid O Alfarouk, Daniel Verduzco, Marilyn M Bui, Robert J Gillies, Muntaser E Ibrahim, Joel S Brown, Robert A Gatenby

**Affiliations:** 1 H. Lee Moffitt Cancer Center, 12902 Magnolia Drive, Tampa, FL 33612, USA; 2 Unit of Diseases and Diversity, Dept. of Molecular Biology, Institute of Endemic Diseases, University of Khartoum, Medical Campus, P. O. BOX 102, Khartoum, Sudan; 3 University of Illinois at Chicago, Chicago, IL, USA

**Keywords:** Darwinian dynamics, ER, Breast cancer, Selection, Phenotypic prediction, Vasculature, Hecrosis

## Abstract

**Background:**

Breast carcinoma can be classified as either Estrogen Receptor (ER) positive or negative by immunohistochemical phenotyping, although ER expression may vary from 1 to 100% of malignant cells within an ER + tumor. This is similar to genetic variability observed in other tumor types and is generally viewed as a consequence of intratumoral evolution driven by random genetic mutations. Here we view cellular evolution within tumors as a classical Darwinian system in which variations in molecular properties represent predictable adaptations to spatially heterogeneous environmental selection forces. We hypothesize that ER expression is a successful adaptive strategy only if estrogen is present in the microenvironment. Since the dominant source of estrogen is blood flow, we hypothesized that, in general, intratumoral regions with higher blood flow would contain larger numbers of ER + cells when compared to areas of low blood flow and in turn necrosis.

**Methods:**

This study used digital pathology whole slide image acquisition and advanced image analysis algorithms. We examined the spatial distribution of ER + and ER- cells, vascular density, vessel area, and tissue necrosis within histological sections of 24 breast cancer specimens. These data were correlated with the patients ER status and molecular pathology report findings.

**Results:**

ANOVA analyses revealed a strong correlation between vascular area and ER expression and between high fractional necrosis and absent ER expression (R^2^ = 39%; p < 0.003 and R^2^ = 46%; p < 0.001), respectively). ER expression did not correlate with tumor grade or size.

**Conclusion:**

We conclude that ER expression can be understood as a Darwinian process and linked to variations in estrogen delivery by temporal and spatial heterogeneity in blood flow. This correlation suggests strategies to promote intratumoral blood flow or a cyclic introduction of estrogen in the treatment schedule could be explored as a counter-intuitive approach to increase the efficacy of anti-estrogen drugs.

## Background

Estrogen (17β-estradiol) is a circulating steroid hormone that binds to intracellular estrogen receptors (ER) after passively diffusing through the plasma membrane (Thomas et al. [1]). Estrogen frequently plays a crucial role in breast tumorigenesis by promoting cellular proliferation, and decreasing apoptosis ([2-4]; Russo et al. [5]; Yager et al. [6]). ER expression in breast cancers is used as a prognostic and predictive tool that reliably correlates with the clinical progression of disease and its response to hormonal therapies.

Although the ER status of breast carcinomas is typically expressed as simply positive or negative, there is frequently considerable heterogeneity of ER expression among cells of the same tumor. In fact, typical classification of a tumor as ER positive requires only 1% of the cells expresses ER (Hammond et al. [7]). There is now evidence [8,9] that the prevalence of ER expression within cells in the same tumor correlates with the degree and duration of response to anti-estrogen therapy.

Our goal here is to investigate the evolutionary and ecological forces that govern heterogeneity of ER expression in breast cancers. Recent studies have demonstrated substantial heterogeneity in cells within the same tumor as a result of intratumoral evolution [10-14]. Generally, this heterogeneity is viewed as a genetic process in which stochastic mutations generate new populations in an unpredictable if not chaotic process. We note, however, that genetic changes are simply one component of evolution and that intratumoral Darwinian dynamics emerge fundamentally from environmental selection forces that promote phenotypic (not genotypic) adaptations [15]. Furthermore, we acknowledge that a large body of work exists which addresses the complex dynamics of ER expression in vitro [16,17] and in vivo (Shipitson et al. [18]). We embrace these works and do not suggest that phenotypic adaptation alone is sufficient explain variation in ER expression.

Instead, we propose that intratumoral cellular heterogeneity represents a predictable process driven by variations in environmental selection forces leading to predictable and reproducible adaptive strategies. The most obvious source of environmental selection is blood flow which, in most cancers, is spatially and temporally heterogeneous resulting in regions of necrosis in poorly perfused regions.

We propose that ER expression will be observed if it provides an adaptive advantage. Specifically, we propose that ER will be expressed only when estrogen is present in the microenvironment. When estrogen is absent, ER expression represents a needless expenditure of resources and will be selected against. Since the source of estrogen in the breast is typically (although not always) interstitial fluid and moves from the vessels into the cell by a simple reaction diffusion model identical to oxygen, nutrients, etc. [19], we propose the hypothesis that ER + cells will be found in regions of high blood flow while ER- cells will be present in regions of poor blood flow. This results in the prediction that the prevalence of ER + cells will generally follow the distribution of blood flow. To test this hypothesis we examined regional distribution of ER + and ER- cells compared to vascular density and regional necrosis within 24 clinical breast cancers of variable ER status and tumor grade.

## Methods

### Sample selection and collection

Twenty-four (24) clinically identified breast cancer cases were selected via pathology report reviews by a board certified pathologist (MMB) with the approval of the University of South Florida Institutional Review Board and the Moffitt Cancer Center Scientific Review Committee. Data for each case include the pathologist’s estimation of percent ER + cells, ER stain intensity, and the semi-quantitative Allred score [20] and histological score. The cases cover a wide spectrum of diagnostic stages including ductal carcinoma in situ (DCIS) (n = 11), invasive ductal carcinoma Nottingham Grade I (n = 4), Grade II (n = 4) and Grade III (n = 5). Similarly, the ER status, based on the pathology report, ranged from 0- 100% positive and the Allred and histological score were used to create four classification ranges. The Allred score is the sum of a proportion score reflecting the percentage of positive-staining tumor cells (0, none; 1, 1⁄100; 2, 1⁄100 to 1⁄10; 3, 1⁄10 to 1⁄3; 4, 1⁄3 to 2⁄3; and 5, >2⁄3) and an intensity score representing the average intensity of positive tumor cells (0, none; 1, weak, 2, intermediate; and 3, strong). The proportion and intensity scores are added to obtain a total score, which ranges from 0 to 8. (Harvey [21]). The H score is a combination of staining intensity and extent according to the following formula: H score = 1 ×% of tumor cells with weak staining + 2 ×% of tumor cells with moderate staining + 3 ×% of tumor cells with strong staining, resulting in a total score of 0 – 300 (Elston [22]). Although the ER intensity, Allred and H-Scores were variable for the ER + cases used to measure the vascular density and necrotic area, the percentage of ER positive cells in the positive cases was always greater than 90%. This fact made it challenging to assess the spatial distribution of ER positivity in these cases. An additional five cases were selected with <60% ER positivity and used to specifically evaluate the spatial distribution of ER positivity with respect to vasculature.

The hematoxylin and eosin (H&E) stained sections used for diagnosis from each identified case were retrieved from the department archives and confirmed by the study pathologist (MMB). The blocks identified to have sufficient material and most representative of each case was retrieved from the Cancer Center archives for the purposes of these studies.

### Histology

For each of the 24 blocks selected for this study, serial unstained sections were cut at a thickness of 4 μm using standard microtomy practices and placed on charged glass slides. The order in which the sections were cut was recorded. The first section was stained with H&E, using standard histological technique. The subsequent serial sections were stained using a mouse monoclonal antibody that reacts to CD34, (#CMA334, Cell Marque, Rocklin, CA) at the stock prediluted concentration and mouse monoclonal ER-β (#ab5786, Abcam, Cambridge, MA) at 1:250 dilution and monoclonal antibody VEGF VG1 (#M7273, Dako, Carpinteria, CA) at 1:500 dilution. These slides were incubated for 16 minutes at room temperature The Ventana OmniMap anti-mouse secondary antibody was incubated for 12 min. The Ventana ChromoMap kit detection system was used according to the kit protocol and slides were then counterstained with hematoxylin. Appropriate positive and negative controls were used. Slides were covered with #1.5 thick cover glass.

### Image acquisition

Whole slide images (WSI) were produced using an Aperio (Vista, CA, USA) ScanScope XT digital slide scanner with a 20×/0.75NA lens. Using the Basler tri-linear array detection, stitching was minimized and the time to scan for most WSIs did not exceed five minutes. Digital WSIs were retained on servers housed within the Moffitt Network Operations Center and accessible on any networked computer via the password protected Spectrum (Aperio) database.

### Image analysis

#### H&E segmentation

The commercially available Genie histology pattern recognition platform (Aperio) was trained to classify regions of interest within each of the 13 invasive H&E stained samples. DCIS was analyzed separately to account for the central comedo necrosis common to this non-invasive stage. By manually selecting regions of necrosis, viable tumor and other tissues (including, but not limited to: skin, adipose tissue, and normal margins) the software was trained with the following settings (1000 iterations of uniform distribution with 0.01 as a regularization parameter over 20 stage iterations with eight and three iterations per first and second stage, respectively). Application of this training set over the entire WSI for each patient allowed for computationally derived region segmentation. Each case was carefully quality controlled by a board certified pathologist (MMB).

#### Vasculature identification and quantification

The CD34 stained slides were segmented by the region classification methods described above. Furthermore, the CD34 positive vessels were identified using the Aperio vasculature algorithm with the following settings (Lumen and closed vessels including incomplete vessels with filtering = 2; low = 160; high =210; with stain components .27, .57 and .78 [RGB]). This algorithm was used to export the quantified values for vessel perimeter, area and lumen area.

#### ER identification and quantification

The ER stained slides were segmented by region classification methods using Definiens Tissue Studio (Munich, Germany). The DCIS and invasive tumor components were identified as the regions of interest and each cell was segmented and classified into negative (masked blue), low (yellow), moderate (orange) and high (red) intensity using the following parameters (IHC Thres = 0.5; Thres low/moderate = 0.75; Thres moderate/high = 1.0)

Resultant classification images from the cell identification, segmentation and classification methods were overlain with vessel mask images from CD34 staining using Image Pro Plus v.6.0.1 (Media Cybernetics, Bethesda, MD). These images were used to measure the shortest distance between each cell and the nearest identified vessel.

### Statistical analysis

A partially-hierarchical ANOVA (SYSTAT version 13) analysis was used to test for the effects vessel number, four parameters of vessel size (mean vessel area, mean vessel perimeter, maximum vessel size and mean lumen area) and the percentage of tissue which is necrotic on the tumor grade and ER status of each case. The dependent variables were the different feature data (i.e. vessel number, mean lumen area and percentage of necrotic area) and the independent variables were ER status and tumor grade. The 24 cases being analyzed were the samples.

## Results

### Vasculature availability

CD34 positive blood vessels within a manually edited buffer of 300 μm from any tumor cell in all directions were identified and individually quantified for each sample. The metrics collected included the number of vessels in the sample. No correlation between vessel number and ER status was elucidated (R^2^ = 7%; p = 0.689).

**Figure 1:**
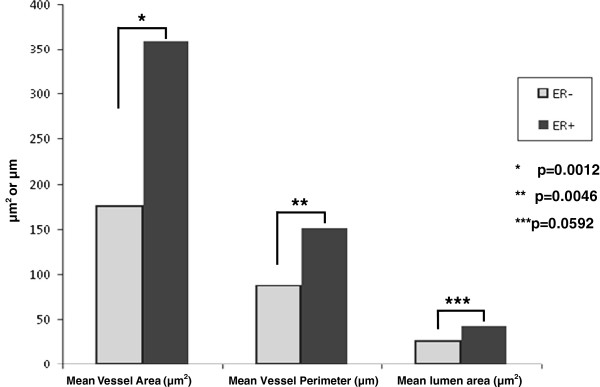
**Vascular quantification.** This graph demonstrates the quantified vascular differences between ER + and ER- samples. Largely, the ER + samples exhibit vessel area, perimeter length and lumen size which are statistically significant to demonstrate increased size as compared to the vessels in ER- samples.

**Figure 2:**
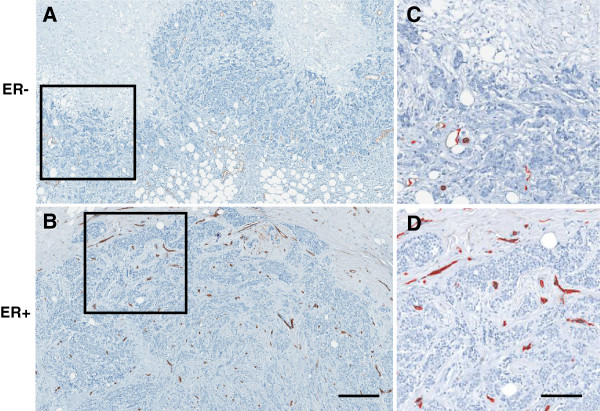
**Representative vasculature images. A)** ER- Grade III invasive breast cancer tumor stains against CD34 with as few as 13 quantified vessels at a region adjacent to the tumor edge. Scale = 800 μm. This may be compared with **B)** ER + CD34 stained grade III tumor which has as many as 84 vessels in the same area as evidenced by **C)** and **D)** which are enlarged views of the inset areas with quantified vessels of each masked in red. Scale bar = 200 μm.

The vessel size (mean vessel area, mean vessel perimeter, maximum vessel area and mean lumen area)) of the blood vessels was much lower in the samples which did not express ER compared to the ER + samples as evidenced in Figure 1 and Table 1. In aggregate the ER- samples exhibited a mean vessel area of 176 μm^2^ compared to 359 μm^2^ in ER + patients (R^2^ = 37%; p = 0.003). The perimeter increased from 87 μm in ER- to 151 μm in ER + (R^2^ = 40%; p = 0.003). Even the maximum vessel area and lumens of the vessels increased from 25 μm^2^ in ER- to 41 μm^2^ in the ER + cohort (R^2^ = 18%; p = 0.059). To reiterate, the mean vessel diameter of the vasculature of the ER + regions was about twice that of the vessels if ER- samples in each of the three measures. Vessels identified in the ER- regions never exceeded a mean area over 300 μm^2^ while 14 of the 18 ER + cases exhibited a mean exceeding 300 μm^2^ with a maximum of 671.4 μm^2^ (Table 2) (R^2^ = 16%; p = 0.08). Vessel size was not found to be correlated with disease progression (p = 0.295). In other words, vessel size was not statistically different in DCIS samples compared to those of grade I, II or III invasive cancers yet the same metrics of vessel size was highly correlated with ER status (Figure 2). Furthermore, the vascular density (vessels/area) was not correlated with ER status or disease progression (p = 0.476).

**Table 1:** Vascular quantification

	**ER-**	**Er+**
Mean vessel area (um2)	175.6	358.5
Mean vessel perimeter (um)	87.1	151.2
Mean lumen area (um2)	24.6	40.7

Quantified values of mean vascularity for ER + and ER- patients.

**Table 2:** Quantification metrics for all patients

**De-ID patient #**	**Er + or ER-**	**Percent ER + cells**	**Stain intensity**	**Allred score**	**Histological score**	**# of vessels per unit area (um2)**	**Maximum vessels area (um2)**	**Mean vessels area (um2)**	**Mean vessels perimeter (um)**	**Mean lumen area (um2)**	**Necrotic area per tumor area**
Patient 1	-	0	0	0	0	2.12E-04	1301	197.3	91.5	47.5	23.1
Patient 2	-	0	0	0	0	7.97E-05	153	63.1	41.4	7.7	69.3
Patient 3	-	0	0	0	0	3.67-04	2124	224.4	107.4	21.2	<.5
Patient 4	-	0	0	0	0	5.19E-05	329	102.8	58.2	8.2	16.2
Patient 5	-	0	0	0	0	1.14E-03	12195	287.9	141.5	34.4	22.4
Patient 6	-	0	0	0	0	1.56E-03	8609	178.0	82.9	28.9	14.9
Patient 7	+	90	1+ to 3+	6	150	6.19E-04	13082	482.8	195.7	72.3	33.7
Patient 8	+	95	1+ to 3+	7	155	4.35E-04	4560	307.4	142.1	44.6	1.1
Patient 9	+	95	1+ to 3+	7	165	8.38E-04	12672	282.1	122.8	40.4	<.5
Patient 10	+	90	2+	7+	170	7.23E-04	14879	347.7	153.3	34.8	22.1
Patient 11	+	100	1+ to 3+	8	200	4.79E-05	1784	368.6	138.2	33.3	4.7
Patient 12	+	95	1+ to 3+	7	240	5.39E-04	6274	267.6	107.4	61.5	<.5
Patient 13	+	100	2 + 0 to 3+	8	260	5.59E-05	7927	671.4	258.4	16.5	5.2
Patient 14	+	90	3+	8	270	9.18E-04	19704	465.8	191.7	23.2	2.5
Patient 15	+	90	3+	8	270	7.66E-04	16094	341.9	130.9	21.7	<.5
Patient 16	+	100	2+ to 3+	8	270	4.67E-04	1712	178.8	98.7	40.4	1.6
Patient 17	+	100	2+ to 3+	8	270	7.27E-04	19485	436.3	174.1	29.3	<.5
Patient 18	+	100	2+ to 3+	8	280	8.26E-04	4379	221.2	110.9	65.8	<.5
Patient 19	+	100	3+	8	300	2.35E-04	1619	172.4	80.6	30.9	3.9
Patient 20	+	100	3+	8	300	8.78E-05	3673	312.2	152.3	17.6	<.5
Patient 21	+	100	3+	8	300	5.03E-04	8258	271.8	115.4	47.7	<.5
Patient 22	+	100	3+	8	300	4.87E-04	17390	373.7	158.7	52.3	<.5
Patient 23	+	100	3+	8	300	7.74E-04	14857	512.3	237.2	58.8	<.5
Patient 24	+	100	3+	8	300	7.03E-04	11188	438.0	156.7	40.9	<.5
Neg control	NA	NA	NA	NA	NA	1.72E-04	2207	116.1	59.3	6.3	NA
Pos control	NA	NA	NA	NA	NA	9.78E-04	11836	345.8	156.3	22.4	NA

Summary of all quantification metrics for all patients.

Increasing mean vessel area is associated with increasing tumor perfusion which might represent potential good prognostic value [23], and increasing survival rate [24], because tumor oxygenation increases survival rate through improving radiation, chemotherapy and reduces metastatic potentiality (Overgaard et al. [25-27]; Jain et al. [28]). These data confirmed that ER-positive tumors have relatively higher average vessel size that might indicate good prognostic value in compare to ER-negative tumors (Teschendorff et al. [29]; [30]).

The spatial distribution of ER positivity with respect to vasculature has been of great interest. Five IDC cases were stained with ER (<60% positivity) were used to specifically evaluate the spatial distribution of ER positivity with respect to identifiable blood vessels. Here the study pathologist (MMB) identified visible vasculature directly from the ER stained slides and from CD34 stained serial sections. Larger vessels were clearly identifiable and demonstrated proximal (<30 μm) ER positivity in 76.5% (26 of 34) visible vessels. Furthermore, for all lesions proximal to vasculature the overall ER positivity was 43.3% and the moderate to strong positivity was 31.5% whereas, conversely, in the lesions distant to vessels ER positivity dropped to 26.3% and the moderate to strong positivity was observed to be 9.3%.

### Necrosis

**Figure 3:**
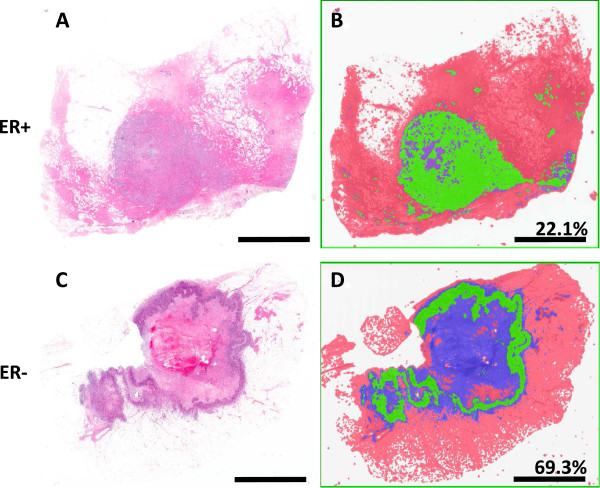
**Invasive necrosis quantification.** Viable tumor is selected via histological pattern recognition software (Genie; Aperio) in green. Other tissues are classified in red and necrotic tissues are classified as blue. In the top panel **A)** and **B)** and ER + sample is presented in comparison to **C)** and **D)** in which an ER- sample is shown. Area of necrosis as a percentage of total tumor area (viable tumor and necrotic region) data for each sample is presented. Scale bar = 5 mm.

Necrosis was once often associated with poor vascularization, subsequent hypoxia and the resultant cell death [31,32]. Other studies have shown that in fact high vascularization is correlated with necrotic zone expansion [33]. In this study necrosis was segmented from the viable tumor and other tissues including normal margins, adipose tissues et cetera. First, it was necessary to segment the patients by diagnosis so central comedo necrosis commonly found in DCIS patients did not over inflate the results of the invasive population. Regardless of the diagnosis, the viable tumor to necrotic area ratio was calculated for each sample (Table 2). Summary statistics were calculated by ER negative and ER positive groups for each diagnostic category. The mean necrosis area in invasive ER- samples was observed to be 24.3%. By contrast, necrosis was quantified to be 4.2% in ER + tumor samples, demonstrating significantly lower necrosis in ER + tumors as compared to ER- tumors (R^2^ = 46%; p < 0.001). Of the ER + invasive samples 8 of 11 had less than 5% necrosis (Figure 3).

**Figure 4:**
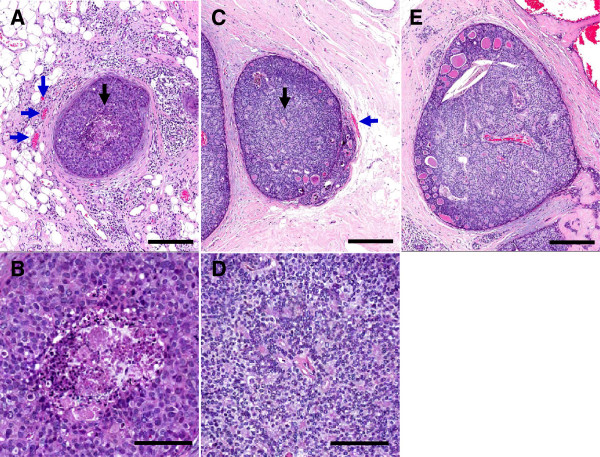
**Ductal carcinoma in situ localization.** This is a DCIS lesion from **A)** an ER- patient with central necrosis. Note the regionally adjacent vessels outside the lesion (blue arrows) and **B)** the enlarged image of the central necrosis localized under the black arrow. **C)** DCIS from an ER + patient without central necrosis despite the size and distance from the center to vasculature; **D)** as illustrated by the lack of necrosis in the enlarged region uder the black arrow. **E)** Shows a large (>1 mm) DCIS sample with interior vasculature. This sample does not exhibit necrosis and this patient is ER+. Top row scale bars = 250 μm; bottom row scale bars = 100 μm.

Similarly, the DCIS ER- group exhibited 21.3% necrosis area per total tumor area while ER + DCIS cases were quantified to contain 4.4% necrosis. Of the DCIS ER + samples 6 of 8 had less than 5% necrosis. These data demonstrate in this sample group that ER-negative tumors have higher necrotic core area relative to viable tumor area. Furthermore, the amount of necrosis in DCIS samples was not found to be dependent on the size of the DCIS or the availability of vasculature outside of the basement membrane of the duct itself. Initially, we hypothesized in DCIS increased necrosis would correlate with ductal size and in turn the distance from the center of the gland to vascular resources. This was not the case in our results. The average diameter of ER- DCIS with central necrosis was 668 μm. The average diameter of ER + DCIS without central necrosis was 702 μm. Examples of the levels of necrosis in ER = and ER- samples are available in Figure 4. Also of interest, in a single case vasculature was observed inside the DCIS. This case was not found to contain necrosis and was ER + (Figure 4E). Finally, adjacent normal breast tissues were also investigated to understand whether or not the vasculature of adjacent normal tissues correlated with the ER positivity in the nearest lesions. The vascularization studies were performed on ten samples which we either 0% ER + (n = 5) or >90% ER + (n = 5). However, there were no observable differences between the vessel features in the adjacent normal tissues.

## Discussion

**Figure 5:**
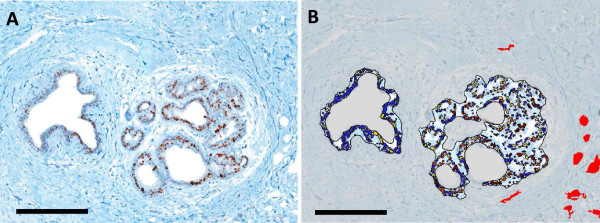
**ER positivity in DCIS relative to vascular localization: A) DCIS lesions stained this ER were B) isolated from the adjacent stroma and the individual cells were segmented and classified as negative (blue), weak (yellow), moderate (orange) and strong (red) stain intensity expression.** Simultaneously CD34 serial sections were used to identify, isolate and mask (red) vessels. Together this image demonstrates an overlain image set of ER classification and vessel localization. Cells nearer the vasculature were observed to have stronger ER expression than cells distant from the vessels. Scale bars = 250 μm.

ER expression is a useful predictive and prognostic biomarker in breast cancer, however, is often extremely variable even within the same tumor. Similar to cellular heterogeneity found in other cancers [10-14], this variation in ER staining is a consequence of intratumoral evolutionary dynamics (Figure 5). Here we address the Darwinian dynamics that might govern cellular ER expression. There are two general components of ER evolution. The first, which cannot be answered with our current study, is the variability of ER expression in the same tumor. Can breast cancer cells adjust ER expression (and other growth factor receptors) so that ER + and - cells in the same tumor represent a single generalist population that phenotypically adapts to various environments? Alternatively the ER expression could be a relatively fixed property and then ER + and – cells represent separate, specialist populations. The second component of intratumoral Darwinian dynamics is the environmental factors that are selection forces that define phenotypic fitness. This is the focus of our current work.

Here we propose that spatial heterogeneity in ER expression is the sequela of intratumoral evolution driven not by random mutations but by variations in environmental selection forces and predictable cellular adaptive strategies. We specifically hypothesize that ER expression will correlate with local concentrations of estrogen. Since estrogen diffusion from blood vessels is spatially limited by reaction–diffusion kinetics similar to oxygen and glucose, we predict a correlation between vascularization, necrosis and ER expression (Teschendorff et al. [29]; [30-32]). In order to test cell density as a plausible barrier of diffusion we evaluated the mean cell number per mm^2^ and did not find any significant difference in the 24 samples evaluated. This suggests cell density alone does not correlate with ER status.

Our results do show that ER + tumors are associated with larger blood vessels and a lower percentage of tissue necrosis. It should however be noted that differences in CD34 staining may over or underestimate the vascularity due to tumor-specific alterations in the vascular bed such that not all of the endothethial cells may be appreciated. Our initial prediction that vessel number will increase in ER + samples was not supported. However, the interaction between vessel size and ER status was three times higher than the interactions between tumor grade and ER status and vessel area. Our second hypothesis, that ER status would be inversely correlated with necrosis, was even more strongly supported. This suggests that as ductal carcinoma in situ progresses towards invasion, 1) the larger the vasculature is early in disease progression, the lower the volume of necrosis and 2) if necrosis does not increase with the cancer progression, then ER + cells are more likely to dominate the population.

While the number of patients evaluated in this study is limited, and a larger patient population would be desirable, the number of individual vessels evaluated is on the order of 10^3^ to 10^4^ per patient. For this reason, our results indicate statistical significance to detect differences between ER positive and negative patients.

Furthermore, the amount of necrosis in DCIS samples was not found to be dependent on the size of the DCIS or the availability of vasculature outside of the basement membrane of the duct itself. Initially, we hypothesized in DCIS increased necrosis would correlate with ductal size and in turn the distance from the center of the gland to vascular resources. This was not the case in our results. Also of interest, in a single case vasculature was observed inside the DCIS. This case was not found to contain necrosis and was ER + (Figure 4E).

Poor vascularization and necrosis was originally hypothesized to be a proxy for hypoxia induced cell death and thus an indicator of low estrogen availability. However, there are a number of plausible explanations (many of which may be responsible in part) why necrotic regions may play a role. In our previous work, we hypothesized that tumor heterogeneity could be predictable similar to that of a riparian zone in a desert environment. Oxygenated phenotypes or relatively highly perfused regions could be equivalent to mesic species and poorly vascularized (distal from a blood supply) would be equivalent to xeric species [15]. In this regard, ER-positive phenotypes are mesic while ER-negatives are xeric phenotypes.

Xeric habitats are formed by evaporation of water and accumulation of salt which results in salty soil that select for xeric species. In our scenario, hypoxic phenotypes may be shaped by a depletion of nutrients, oxygen and metabolites. That is why ER-negative cells may be adapted to tissue of poor vascularization and higher necrosis. Of course toxification (i.e. salty soil) in such an environment may be another plausible consideration. Regardless, this unavailability of estrogen is one reasonable explanation for estrogen-independent tissue selection. This may be a testable hypothesis in vitro or using techniques including laser capture microdissection to isolate specific regions of high vascularity within patient tumors and evaluating the estrogen concentrations. This is a key future direction for this research.

A significant limitation of our analysis is our inability to measure temporal variations in blood flow. That is, the cyclical and random variations in blood flow which have been extensively observed. These variations will result in temporal variations in estrogen concentration which could alter ER expression. This imprecise link of vascular density and blood flow could result in similar variations in the correlation between vascular density and ER expression.

## Conclusion

In conclusion, we find ER expression and metrics of vascular density and blood flow in 24 clinical breast cancers show direct correlations between ER + tumors and blood vessel size and inverse correlation with necrosis consistent with predictions. This correlation, if confirmed, suggest strategies to promote intratumoral blood flow could be explored as a somewhat counter-intuitive approach to increase the efficacy of anti-estrogen drugs.

Due to natural selection, ER- tumors could be evolving in a way that resists (adapts to) the absence of estrogen. As a future direction, we hypothesize that anti-estrogen therapy (e.g. Tamoxifen) can select for ER-independent cells. In contrast, cyclic introduction of estrogen may improve survival rate by continually altering, rather than unilaterally shifting, toward an ER- population. In other words, this theory suggests that modulation (and not eradication or extinction of certain population) may prove to be an advantageous treatment strategy.

Furthermore, it may be possible that ER + cells cluster around vasculature and effectively act as a barrier. While this is a future direction of this research and has not yet been tested, it may explain how both populations coexist spatially in a single tumor. More interestingly, it may also be possible that this spatial pattern keeps ER- cells farther from blood vessels where they might enter the bloodstream and form metastatic tumors. ER- may be more prone to metastasize if ER + cells are less successful invading novel tissues. Future work should determine the spatial relationships around vasculature and the propensity for each population to metastasize. This more in depth assessment of regional distributions of ER + and ER- cells will be important to understand whether heterogeneous ER staining correlates directly with vessel distribution (i.e. whether ER + cells congregate nearer to the vessels within a tumor).

In summary, we conclude that ER status is selected for given vascular availability which could have meaningful and exploitable therapeutic decision making implications.

## Consent

Written informed consent was obtained via Institutional Review Board from the University of South Florida approval for the use of patient information for the publication of this report and any accompanying images.

## Abbreviations

ER: Estrogen receptor; ER-: Estrogen receptor negative; ER+: Estrogen receptor positive; DAB: 3,3’ diaminobenzidine (stain); DCIS: Ductal carcinoma in situ; H&E: Hematoxylin and eosin; IDC: Intraductal carcinoma; IHC: Immunohistochemistry; μm: Micron; WSI: Whole slide images; NA: Numerical aperture; RGB: Red, green, blue; MMB: Marilyn M. Bui (author and pathologist).

## Competing interests

The authors declare that they have no competing interests.

## Authors’ contribution

MCL and KOA conceived of the study, designed the experiments and drafted the manuscript. MCL carried out the image acquisition and analysis studies. MMB selected the patient’s for study participation and participated in many aspects of the pathological analysis. MCL, DV, RJG and MEI participated intellectually in the design and framing of the manuscript. MCL, JSB and RAG provided substantial intellectual direction and participated in experimental design, data analysis and drafting of the manuscript. All authors read and approved the final manuscript.

## Pre-publication history

The pre-publication history for this paper can be accessed here:

http://www.biomedcentral.com/1471-2407/14/279/prepub
